# Heterozygous *Spink1* Deficiency Promotes Trypsin-dependent Chronic Pancreatitis in Mice

**DOI:** 10.1016/j.jcmgh.2024.05.009

**Published:** 2024-05-18

**Authors:** Alexandra Demcsák, Miklós Sahin-Tóth

**Affiliations:** Department of Surgery, University of California Los Angeles, Los Angeles, California

**Keywords:** Acute Pancreatitis, Cerulein, Chronic Pancreatitis, Trypsin, Trypsin Inhibitor, Trypsinogen

## Abstract

**Background & Aims:**

Heterozygous *SPINK1* mutations are strong risk factors for chronic pancreatitis in humans, yet heterozygous disruption of mouse *Spink1* yielded no pancreatic phenotype. To resolve this contradiction, we used CRISPR/Cas9-mediated genome editing to generate heterozygous *Spink1*-deleted mice (*Spink1-KO*^het^) in the C57BL/6N strain and studied the effect of this allele in trypsin-independent and trypsin-dependent pancreatitis models.

**Methods:**

We investigated severity of acute pancreatitis and progression to chronic pancreatitis in *Spink1-KO*^het^ mice after transient (10 injections) and prolonged (2 × 8 injections) cerulein hyperstimulation. We crossed *Spink1-KO*^het^ mice with *T7D23A* and *T7D22N,K24R* mice that carry strongly autoactivating trypsinogen mutants and exhibit spontaneous chronic pancreatitis.

**Results:**

Prolonged but not transient cerulein stimulation resulted in increased intrapancreatic trypsin activity and more severe acute pancreatitis in *Spink1-KO*^het^ mice relative to the C57BL/6N control strain. After the acute episode, *Spink1-KO*^het^ mice developed progressive disease with chronic pancreatitis-like features, whereas C57BL/6N mice recovered rapidly. Trypsinogen mutant mice carrying the *Spink1-KO*^het^ allele exhibited strikingly more severe chronic pancreatitis than the respective parent strains.

**Conclusions:**

Heterozygous *Spink1* deficiency caused more severe acute pancreatitis after prolonged cerulein stimulation and promoted chronic pancreatitis after the cerulein-induced acute episode, and in two strains of trypsinogen mutant mice with spontaneous disease. In contrast, acute pancreatitis induced with limited cerulein hyperstimulation was unaffected by heterozygous *Spink1* deletion, in agreement with recent observations that trypsin activity does not mediate pathologic responses in this model. Taken together, the findings strongly support the notion that loss-of-function *SPINK1* mutations in humans increase chronic pancreatitis risk in a trypsin-dependent manner.

**Summary**

Heterozygous loss-of-function variants in the *SPINK1* gene that encodes a trypsin inhibitor are strong risk factors for chronic pancreatitis in humans. This study demonstrates that heterozygous loss of SPINK1 in preclinical mouse models promotes pancreatitis onset and progression in a trypsin-dependent manner.

The pancreas secretes a variety of protease precursors, which become activated in the small intestine and catalyze digestion of dietary proteins and peptides.^1^ The most abundant protease proenzyme is trypsinogen, which has the unique ability to undergo so-called autoactivation; a self-amplifying, bimolecular reaction of trypsin activating trypsinogen.^2^ Trypsinogen autoactivation inside the pancreas can trigger the inflammatory disorder pancreatitis. To guard against unwanted intrapancreatic trypsin activity, acinar cells produce the serine protease inhibitor Kazal type 1 (SPINK1), a 6.2-kDa secretory trypsin inhibitor. SPINK1 represents approximately 0.1% to 0.8% of the total pancreatic juice protein, which is equivalent to about 2% to 13% of the trypsinogen content in molar terms.^2^ SPINK1 binds to the major human and mouse trypsin isoforms with high affinity, and forms non-covalent complexes.^3^^,^^4^ The equilibrium dissociation constant (*K*_D_) of the binding reaction is in the picomolar range.^3^^,^^4^ Curiously, post-translational tyrosine sulfation of human trypsins weakens inhibitor binding.^3^ In the presence of excess trypsin, the inhibitory complexes slowly dissociate due to proteolytic inactivation of SPINK1, a phenomenon termed temporary inhibition.^4^ In the pancreas, the inhibition of trypsin by SPINK1 not only neutralizes any potential harmful effects of the protease, but also prevents trypsinogen autoactivation. Pancreatitis may occur when intrapancreatic trypsin activity overwhelms SPINK1 reserves, and trypsinogen autoactivation can proceed unimpeded (Figure 1).

**Figure 1:**
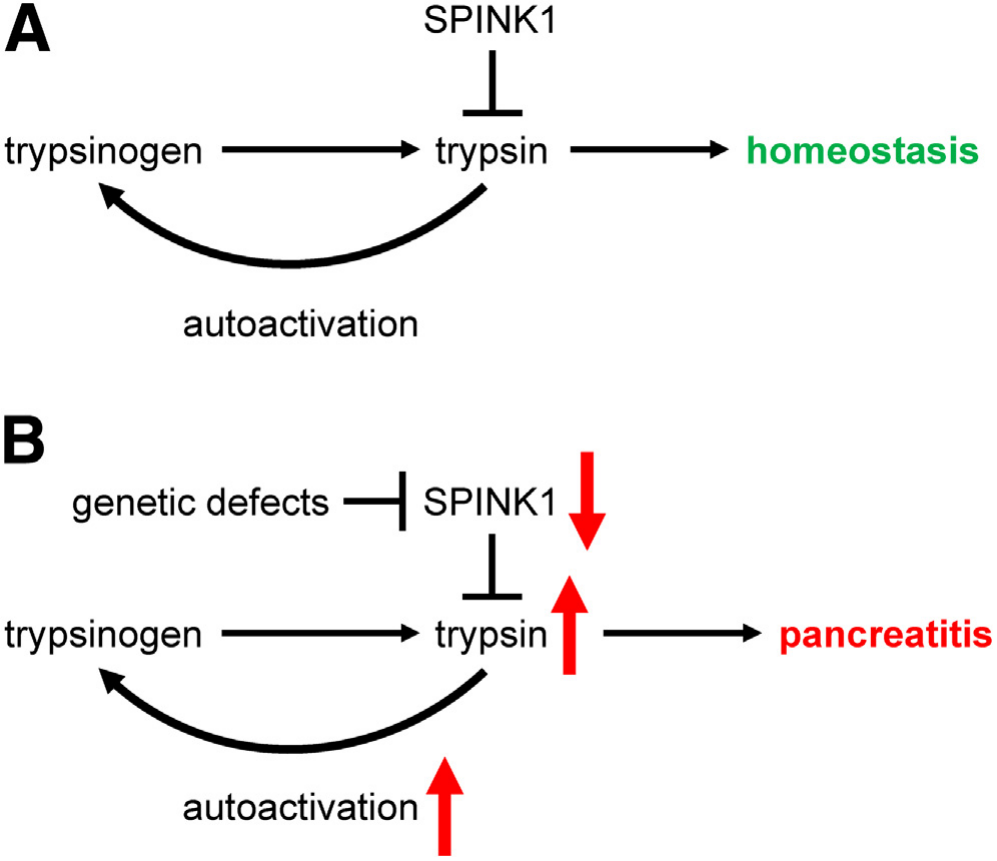
**Role of SPINK1 in pancreas health.** (*A*) SPINK1 maintains homeostasis through trypsin inhibition and control of trypsinogen autoactivation. (*B*) Loss-of-function *SPINK1* mutations reduce protective SPINK1 levels, leading to increased trypsinogen autoactivation, higher intrapancreatic trypsin activity, and increased risk of pancreatitis.

Evidence that SPINK1 maintains pancreas health came from human genetic studies that demonstrated that loss-of-function variants in *SPINK1* strongly increase the risk for chronic pancreatitis (CP).^2^^,^^5^^,^^6^ Two relatively frequent variants (p.N34S and c.194+2T>C) and numerous rare variants have been described and replicated in several cohorts.^2^^,^^5^, ^6^, ^7^, ^8^, ^9^ Although the mechanism of action of the p.N34S variant is still unsettled, the splice-site variant c.194+2T>C offered compelling evidence for a loss-of-function disease mechanism. Multiple studies demonstrated that c.194+2T>C caused exon skipping and loss of *SPINK1* mRNA expression.^10^, ^11^, ^12^ Many rare *SPINK1* variants also support the loss-of-function pathogenic model. These include promoter variants, translation initiator methionine variants, signal peptide variants, splice-site variants, frame-shift variants, and missense variants that impair SPINK1 secretion.^2^ It is interesting to note that almost all pathogenic *SPINK1* variants described to date diminish expression and/or secretion of the inhibitor. Variants that affect trypsin inhibitory activity are exceptions.^3^ In the majority of cases, *SPINK1* variants are detected in the heterozygous state. Homozygosity is relatively rare and can be considered disease-causing.

Mice express a single orthologue of human SPINK1; the secreted forms of the 2 proteins are 63% identical in their amino-acid sequence. Ohmuraya et al described the first mouse model with deletion of the *Spink1* gene (named *Spink3* at the time).^13^ Homozygous *Spink1*-deficient mice died shortly after birth, and acinar cells showed extensive vacuolization apparently due to autophagy. There was spontaneous trypsin activity detectable in isolated acini, which could be inhibited by nafamostat.^14^^,^^15^ Heterozygous mice showed no spontaneous phenotype. Cerulein-induced trypsin activity and acute pancreatitis were unchanged relative to wild-type littermates. The unexpected finding that heterozygous *Spink1*-deleted mice exhibited no change in severity of cerulein-induced acute pancreatitis was later confirmed by Rodger Liddle’s group.^16^ Thus, heterozygous loss of *Spink1* in mice does not replicate the human condition where heterozygous *SPINK1* variants have a strong effect on CP risk.

Transgenic overexpression of a rat *SPINK1* paralog was found to protect against cerulein-induced acute pancreatitis, cerulein-induced CP, and CP elicited by pancreatic overexpression of interleukin-1β.^17^, ^18^, ^19^ These observations were surprising because the experimental models employed were not trypsin-dependent. Furthermore, the findings were difficult to reconcile with the results of the *Spink1* deletion studies. The Ohmuraya laboratory generated a mouse model where the human *SPINK1* gene was integrated into the X chromosome in homozygous *Spink1*-deleted mice.^20^ Human *SPINK1* rescued the lethal phenotype of *Spink1*-deficient mice, and due to random inactivation of one of the X chromosomes, showed a mosaic pattern of expression in females. Acinar cells not expressing SPINK1 suffered extensive vacuolization and died, whereas SPINK1-expressing acinar cells were normal. Even though the mice developed CP as a result of the mosaic cell injury, the human relevance of this elegant model is uncertain. Finally, preliminary data published in 2020 suggested that mice with the c.194+2T>C *Spink1* mutation might develop CP, but full characterization of this potentially exciting model is still lacking.^21^

Recent years saw a surge in the development of genetically engineered mouse models where increased activation of mutant trypsinogens drive pancreatitis onset and progression.^22^, ^23^, ^24^, ^25^, ^26^, ^27^, ^28^ The availability of these models offers a fresh approach to address the role of heterozygous *Spink1* deficiency in trypsin-mediated CP. In the present study, we resolved the long-existing contradiction between human genetic studies and animal experiments by demonstrating that heterozygous loss of *Spink1* worsens CP in a trypsin-dependent manner.

## Results

### Heterozygous Deletion of Spink1 in C57BL/6N Mice

**Figure 2:**
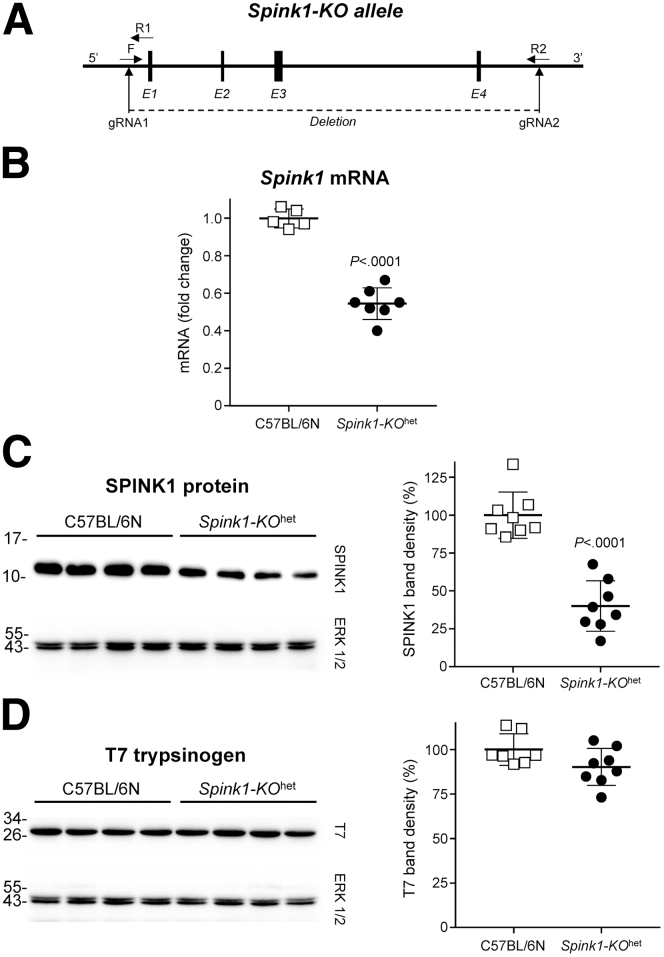
**Effect of heterozygous *Spink1* deletion (*Spink1-KO***^**het**^**) on mRNA and protein expression in the mouse pancreas.** (*A*) CRISPR/Cas9-mediated genome engineering was used to delete the entire *Spink1* gene. E, Exons; F and R, forward and reverse genotyping primers; gRNA, guide RNAs. (*B*) Expression of *Spink1* mRNA in the pancreas of C57BL/6N and *Spink1-KO*^het^ mice was quantified by reverse-transcription quantitative PCR and expressed relative to the average value of the C57BL/6N strain. (*C*) Protein levels of SPINK1 in pancreas homogenates were determined by Western blotting and densitometry. (*D*) Protein levels of mouse cationic trypsinogen (T7) were assessed by Western blotting and densitometry. On Western blots, ERK1/2 was measured as loading control. Representative blots from 2 experiments are shown. Densitometry results are expressed as percent of the average band intensity of the C57BL/6N strain. Individual data points with mean and standard deviation are shown. The difference of means between the groups was analyzed by 2-tailed unpaired *t*-test.

We used CRISPR/Cas9 technology to delete the entire *Spink1* gene in the C57BL/6N mouse genome (Figure 2*A*). Mice heterozygous for the deletion allele (*Spink1-KO*^het^) showed no phenotypic or behavioral changes, bred normally, and pancreas histology was indistinguishable from that of C57BL/6N control mice, up to 6 months of age. Using reverse-transcription quantitative polymerase chain reaction (RT-qPCR) and Western blotting, we confirmed that *Spink1* mRNA (Figure 2*B*) and protein (Figure 2*C*) levels in the pancreas of *Spink1-KO*^het^ mice were reduced by about 50% relative to C57BL/6N control mice, while expression of cationic trypsinogen (isoform T7) was unchanged (Figure 2*D*). We also replicated the published observation that homozygous deletion of *Spink1* resulted in complete pancreas destruction, severe runting, and early death (not shown).^13^^,^^16^

### Cerulein-induced Acute Pancreatitis in Spink1-KO^het^ Mice

**Figure 3:**
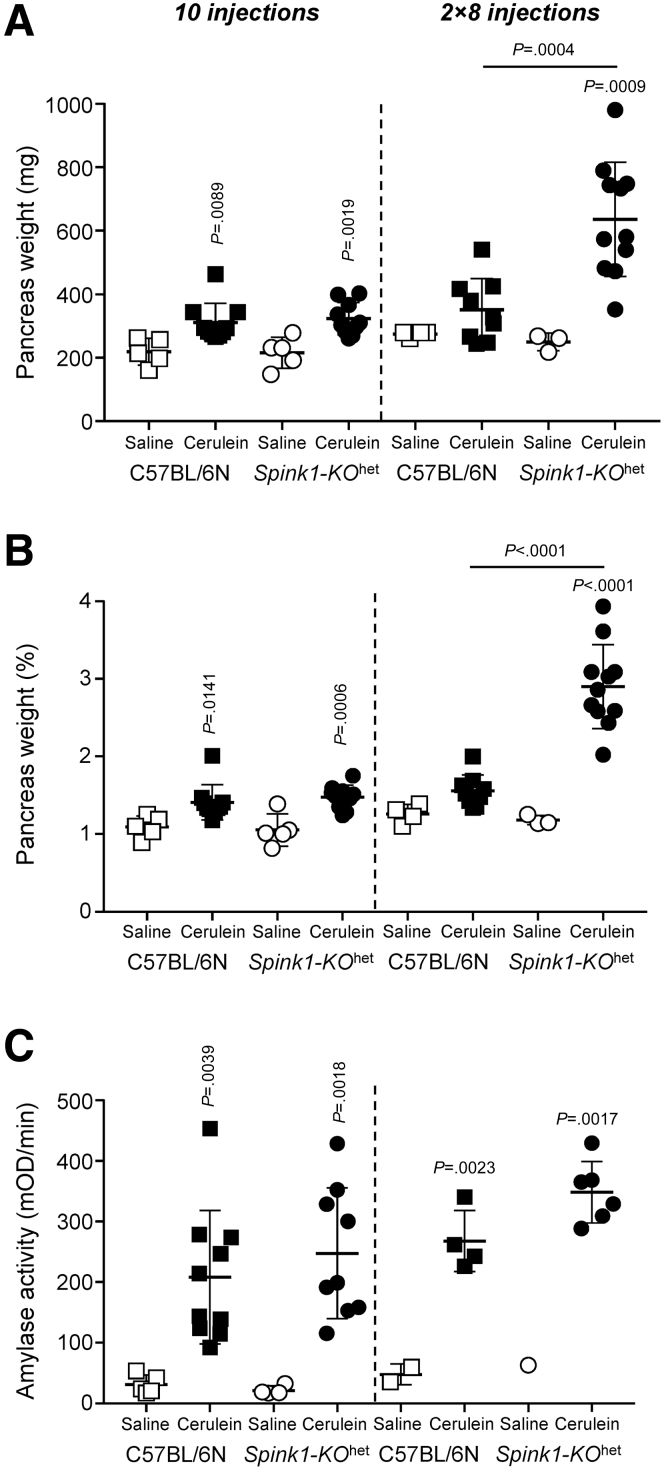
**Cerulein-induced acute pancreatitis in *Spink1-KO***^**het**^**mice.** C57BL/6N and *Spink1-KO*^het^ mice were treated with either transient (10 hourly injections, *left panels*) or prolonged (2 × 8 hourly injections, *right panels*) hyperstimulation with cerulein. Control mice were given saline injections. Animals were euthanized 1 hour (10 injections) or 30 minutes (2 × 8 hourly injections) after the last injection. (*A*) Pancreas weight in mg units. (*B*) Pancreas weight expressed as percent bodyweight. (*C*) Plasma amylase activity. Individual data points with mean and standard deviation are shown. The difference of means between the groups was analyzed by 1-way analysis of variance followed by Tukey’s post-hoc test.

**Figure 4:**
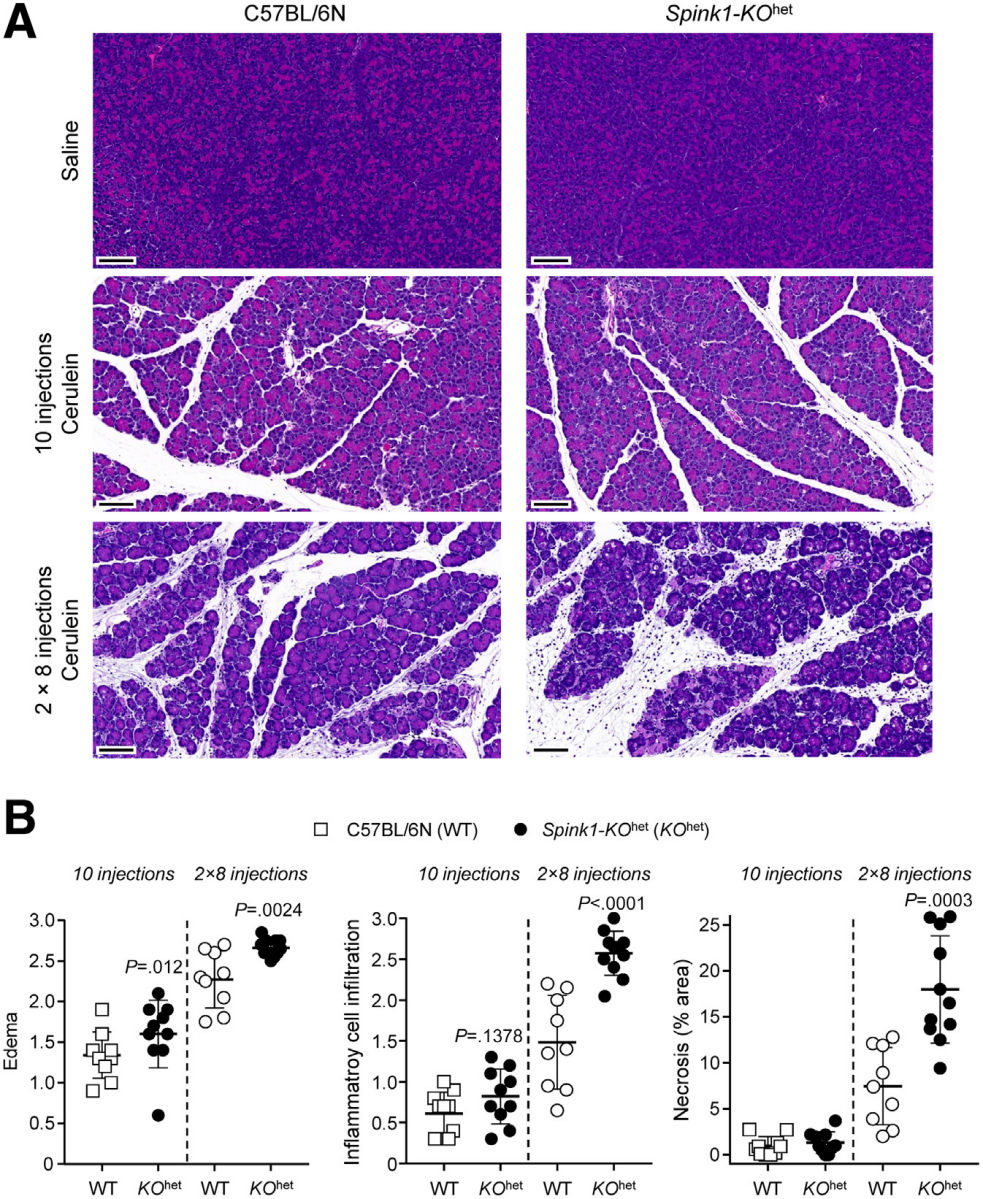
**Histology of cerulein-induced acute pancreatitis in *Spink1-KO***^**het**^**mice.** C57BL/6N and *Spink1-KO*^het^ mice were treated with either transient (10 hourly injections) or prolonged (2 × 8 hourly injections) hyperstimulation with cerulein. Control mice were given saline injections. Animals were euthanized 1 hour (10 injections) or 30 minutes (2 × 8 hourly injections) after the last injection. (*A*) Representative hematoxylin-eosin-stained pancreas sections. Scale bars are 100 μm. (*B*) Histology scoring of pancreas sections from cerulein-treated mice for edema, inflammatory cell infiltration, and acinar cell necrosis. The pancreas of saline-treated mice showed no visible edema, inflammatory cells, or necrosis. Individual data points were graphed with the mean and standard deviation indicated. The difference of means between the groups was analyzed by 2-tailed unpaired *t*-test.

Previous reports found no significant effect of heterozygous *Spink1* deletion on the severity of cerulein-induced acute pancreatitis.^13^^,^^16^ Here, we used hourly injections of cerulein (50 μg/kg) to elicit acute pancreatitis in C57BL/6N and *Spink1-KO*^het^ mice. We tested the effects of transient hyperstimulation (10 hourly injections on the same day) and prolonged hyperstimulation (2 × 8 injections on consecutive days). When compared with saline-injected controls, cerulein treatment induced significant elevations in pancreas weight (Figure 3*A, B*), plasma amylase activity (Figure 3*C*), and histological parameters such as edema, inflammatory cell infiltration, and acinar cell necrosis (Figure 4). With the transient hyperstimulation model, there was no significant difference in disease severity between the 2 strains studied. In contrast, prolonged hyperstimulation with cerulein resulted in significantly higher pancreas weight, histological edema, inflammatory cell infiltration, and necrosis in *Spink1-KO*^het^ mice relative to C57BL/6N controls. Plasma amylase activity was also higher in *Spink1*-deficient animals versus C57BL/6N mice, but the difference did not reach statistical significance.

**Figure 5:**
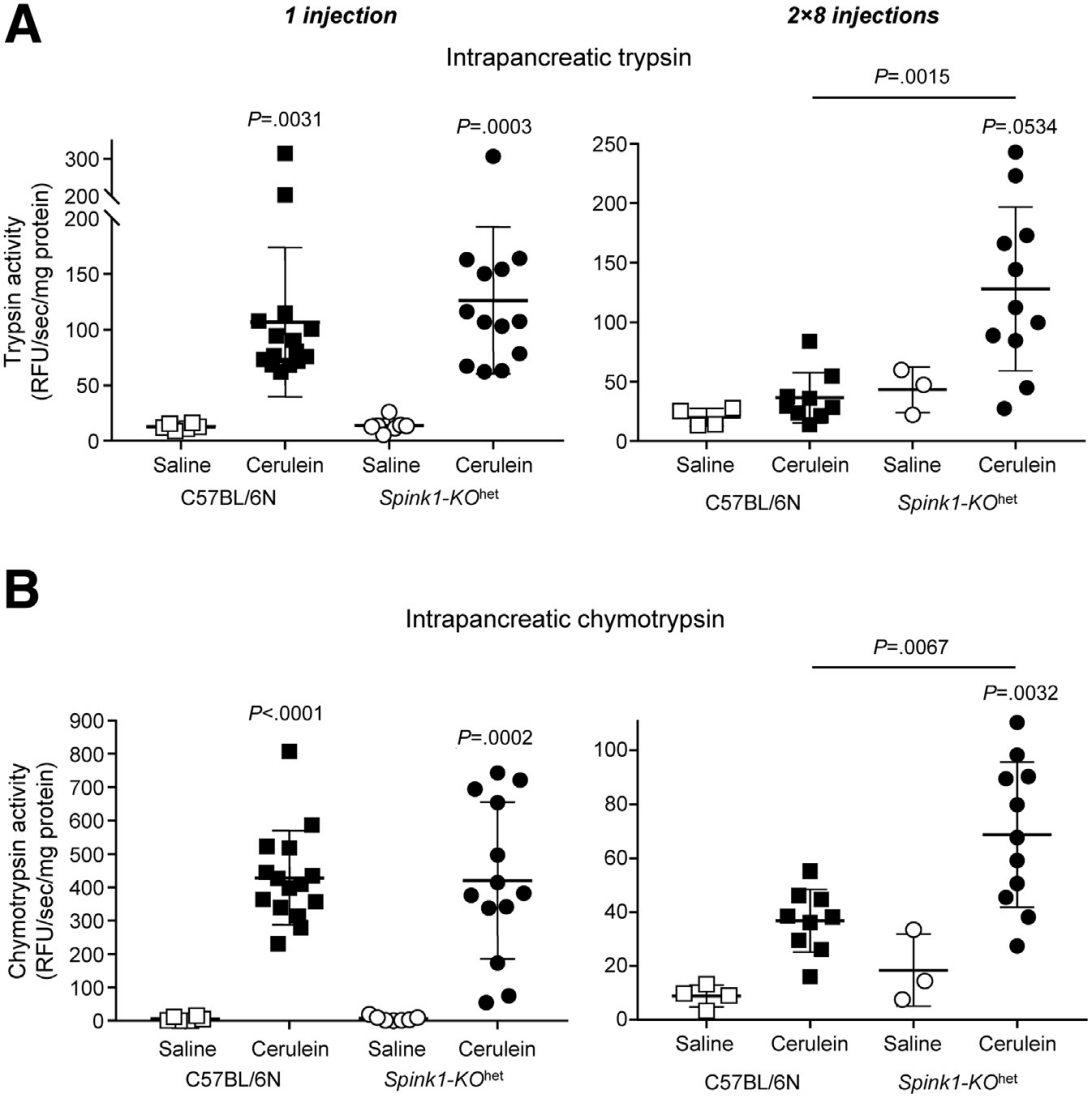
**Cerulein-induced intrapancreatic protease activation in *Spink1-KO***^**het**^**mice.** C57BL/6N and *Spink1-KO*^het^ mice were administered a single injection (left panels) or 2 × 8 hourly injections (*right panels*) of cerulein. Control animals were given saline injections. Mice were euthanized 30 minutes after the single/last injection, the pancreas was homogenized, and protease activities were measured as described in *Methods*. (*A*) Intrapancreatic trypsin activity. (*B*) Intrapancreatic chymotrypsin activity. Individual values with the mean and standard deviation were graphed. The difference of means was analyzed by 1-way analysis of variance with Tukey’s post-hoc test.

We measured cerulein-induced intrapancreatic protease activation 30 minutes after a single cerulein injection and after prolonged (2 × 8 injections) cerulein hyperstimulation (Figure 5). Relative to saline controls, cerulein caused significant increases in trypsin (Figure 5*A*) and chymotrypsin (Figure 5*B*) activity of pancreas homogenates. No appreciable difference was observed in the extent of protease activation between the C57BL/6N and *Spink1-KO*^het^ mice after a single injection of cerulein. In sharp contrast, prolonged hyperstimulation resulted in significantly higher trypsin and chymotrypsin activity in the pancreas of *Spink1-KO*^het^ mice versus C57BL/6N controls.

### Chronic Progression After Cerulein-induced Acute Pancreatitis in Spink1-KO^het^ Mice

**Figure 6:**
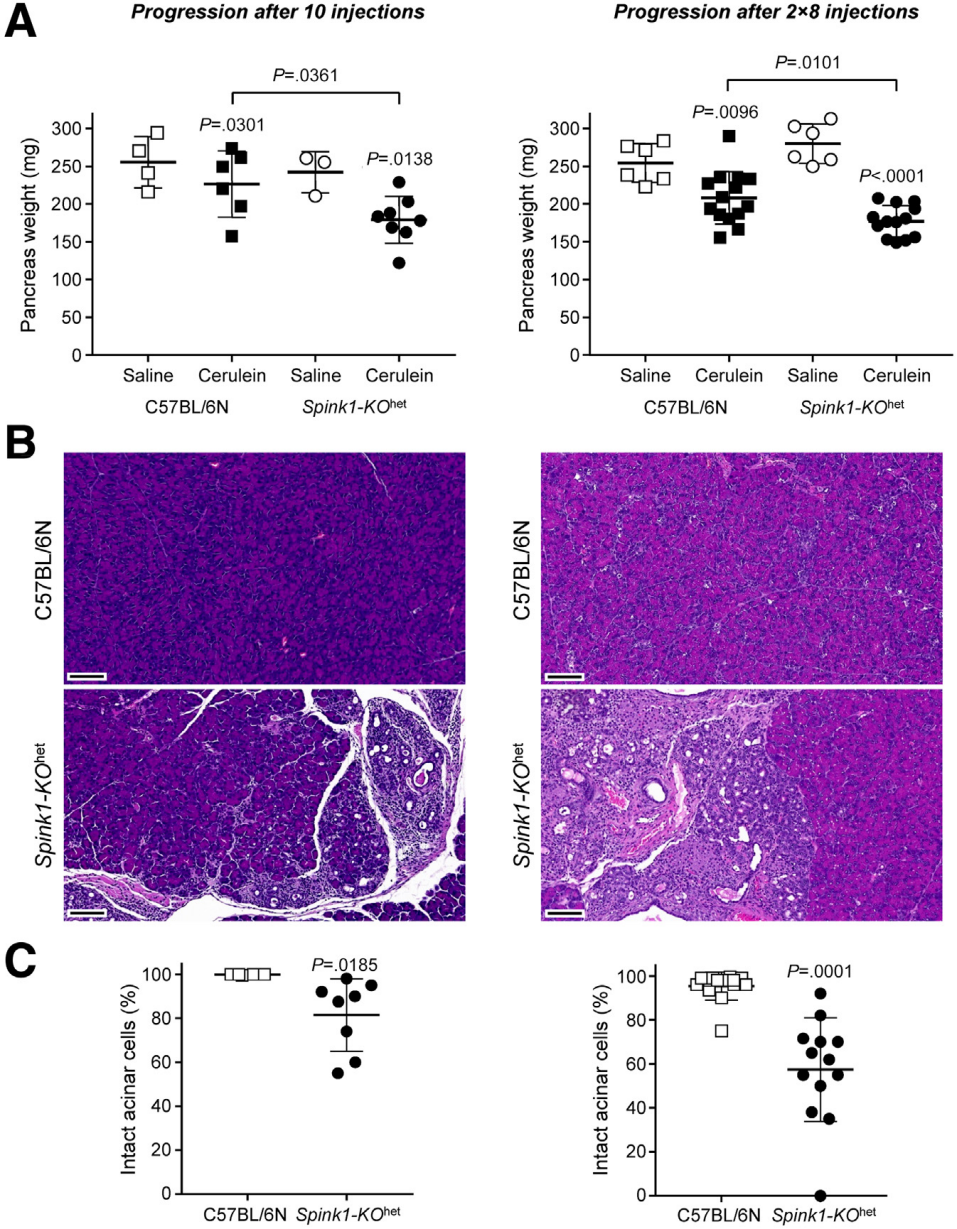
**Disease progression after cerulein-induced acute pancreatitis in *Spink1-KO***^**het**^**mice.** To assess progression after transient hyperstimulation with cerulein, C57BL/6N and *Spink1-KO*^het^ mice were given 10 hourly injections of saline or cerulein and euthanized 1 week later (*left panels*). To evaluate progression after prolonged hyperstimulation with cerulein, mice were given 8 hourly saline or cerulein injections on two consecutive days, and euthanized 3 days later (right panels). (*A*) Pancreas weight. (*B*) Representative hematoxylin-eosin stained sections from cerulein-treated mice. Scale bars are 100 μm. (*C*) Histology scoring for intact acinar cells. Individual values with the mean and standard deviation were plotted. The difference of means between 2 groups and multiple groups was analyzed by 2-tailed unpaired *t*-test and 1-way analysis of variance with Tukey’s post-hoc test, respectively.

**Figure 7:**
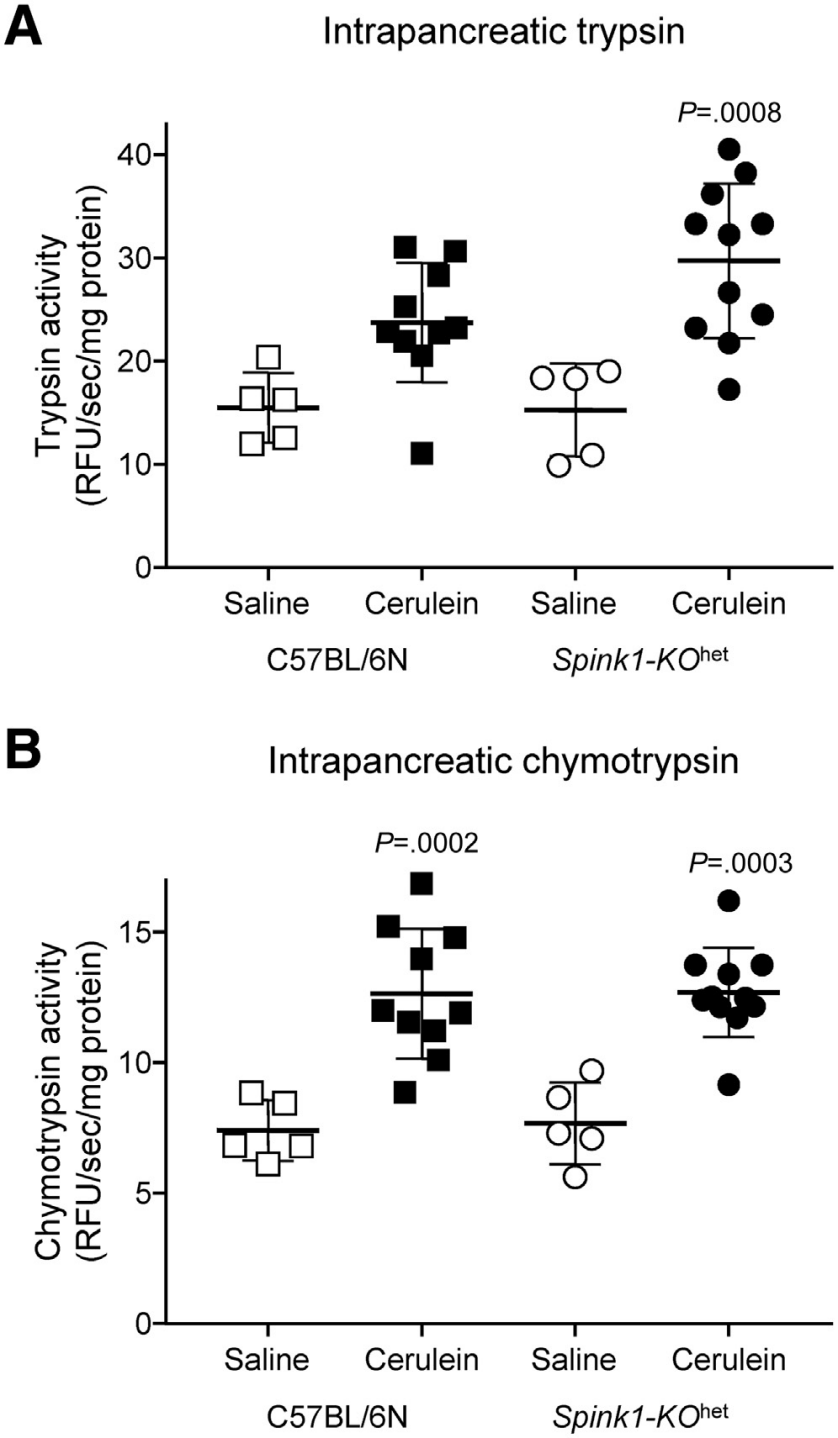
**Intrapancreatic protease activity during disease progression after cerulein-induced acute pancreatitis.** C57BL/6N and *Spink1-KO*^het^ mice were given 2 × 8 hourly injections of saline or cerulein on consecutive days and euthanized 3 days later. The pancreas was homogenized, and protease activities were measured as described in Methods. (*A*) Intrapancreatic trypsin activity. (*B*) Intrapancreatic chymotrypsin activity. Individual values with the mean and standard deviation were graphed. The difference of means was analyzed by 1-way analysis of variance with Tukey’s post-hoc test. The *P* values indicate pairwise comparisons between the saline and cerulein groups. The difference between the cerulein groups was not significant.

C57BL/6N mice rapidly recover after a cerulein-induced pancreatitis attack with essentially complete normalization of pancreas histology within a few days.^29^ In contrast, mice carrying trypsinogen mutants exhibit CP-like, progressive, atrophic changes after an acute episode of cerulein-induced pancreatitis, which can be prevented by administration of a small-molecule trypsin inhibitor.^25^^,^^28^, ^29^, ^30^ Thus, progression to CP after an acute attack is driven by trypsin. When acute pancreatitis in *Spink1-KO*^het^ mice was elicited by transient hyperstimulation with cerulein (10 injections), and animals were euthanized a week after the last injection, the pancreas weight was significantly lower than those of C57BL/6N controls (Figure 6*A*) and histological analysis showed areas of acinar atrophy, dilated ducts, and inflammatory cells (Figure 6*B*). Semi-quantitative scoring for intact acini indicated about 20% histological atrophy on average (Figure 6*C*). A similar but more severe outcome was observed when prolonged hyperstimulation was performed according to the protocol by Jensen et al,^31^ where mice were given 8 hourly injections on 2 consecutive days and were euthanized 3 days after the last injection. Again, we observed reduced pancreas weight (Figure 6*A*), somewhat more extensive chronic changes on histology (Figure 6*B*), and more than 40% acinar cell atrophy in *Spink1-KO*^het^ mice (Figure 6*C*). Importantly, chronic progression in *Spink1-KO*^het^ mice was accompanied by a noticeable trend for increased intrapancreatic trypsin activity relative to C57BL/6N mice, whereas chymotrypsin activity was similar in the 2 strains (Figure 7). The findings are consistent with the notion that development of CP after a cerulein-induced acute episode is trypsin-dependent.

### Heterozygous Spink1 Deficiency Increases Severity of Spontaneous Chronic Pancreatitis in T7D23A and T7D22N,K24R Mice

Mouse models carrying rapidly autoactivating cationic trypsinogen mutants develop progressive CP characterized by significant atrophy, diffuse fibrosis, dilated ducts, inflammatory cell infiltrates, and adipose tissue replacement.^22^^,^^28^ In *T7D23A*^het^ mice, the disease typically starts at the age of 3 to 4 weeks, often with a brief episode of acute pancreatitis followed by rapid progression to chronic changes.^22^ In *T7D22N,K24R*^hom^ mice, the age of onset is around 6 weeks and progression to end-stage CP is slower.^28^ To test whether trypsin-dependent CP in these mouse models is affected by heterozygous *Spink1* deficiency, we generated *T7D23A*^het^ × *Spink1-KO*^het^ and *T7D22N,K24R*^hom^ × *Spink1-KO*^het^ crosses.

**Figure 8:**
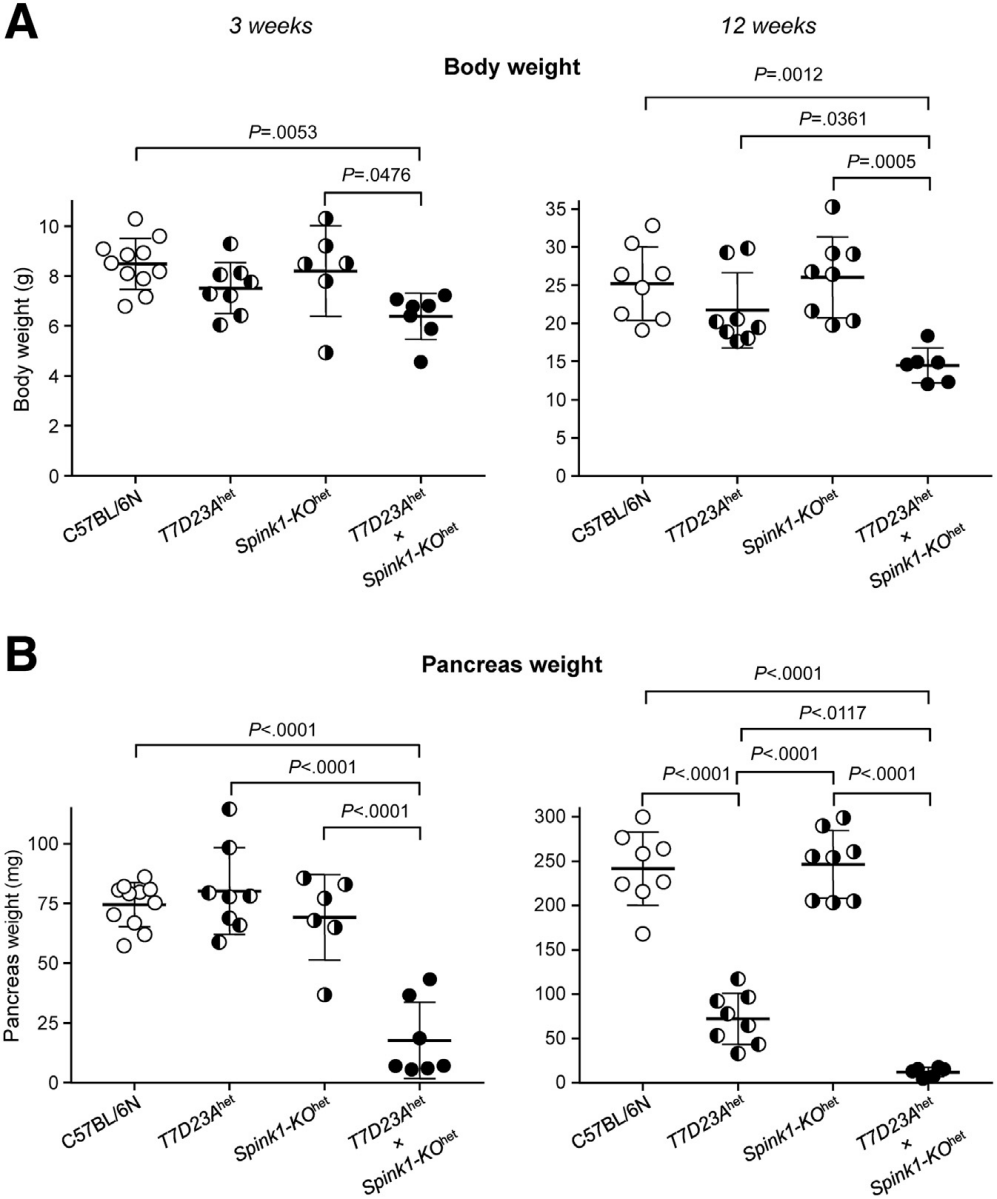
**Effect of heterozygous *Spink1* deletion on chronic pancreatitis severity in *T7D23A* mice.** C57BL/6N, *T7D23A*^het^, *Spink1-KO*^het^, and *T7D23A*^het^ × *Spink1-KO*^het^ mice were euthanized at 3 weeks (*left panels*) and 12 weeks (*right panels*) of age. (*A*) Body weight. (*B*) Pancreas weight. Individual values with the mean and standard deviation were plotted. The difference of means was analyzed by 1-way analysis of variance with Tukey’s post-hoc test.

**Figure 9:**
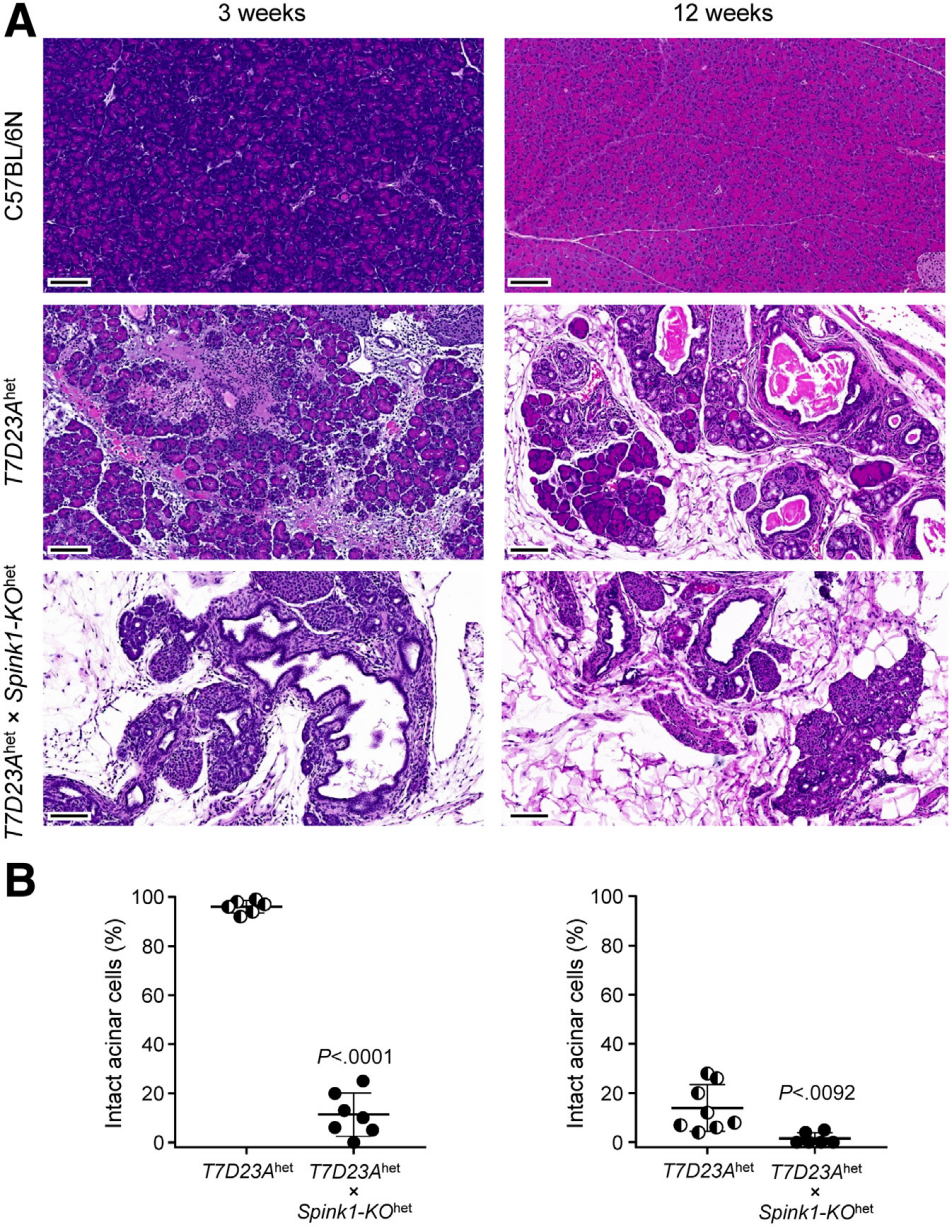
**Histological evaluation of pancreatitis in *T7D23A***^**het**^**× *Spink1-KO***^**het**^**mice.** C57BL/6N, *T7D23A*^het^, and *T7D23A*^het^ × *Spink1-KO*^het^ mice were euthanized at 3 weeks (*left panels*) and 12 weeks (*right panels*) of age. (*A*) Representative hematoxylin-eosin stained pancreas sections. Scale bars are 100 μm. (*B*) Histology scoring for intact acinar cells. Individual values with the mean and standard deviation were plotted. The difference of means between the groups was analyzed by 2-tailed unpaired *t*-test.

When disease severity was analyzed at 3 weeks and 12 weeks, *T7D23A*^het^ × *Spink1-KO*^het^ mice exhibited lower body weight (Figure 8*A*), more severe pancreas atrophy (Figure 8*B*), and more pronounced histological changes (Figure 9*A*) than *T7D23A*^het^ controls, indicating that lower SPINK1 levels caused accelerated progression. At 3 weeks of age, all pancreata from *T7D23A* mice (n = 6) showed signs of acute pancreatitis (edema, inflammatory cell infiltration, small areas of necrosis) without significant acinar cell loss or chronic changes. In contrast, the pancreas from all *T7D23A*^het^ × *Spink1-KO*^het^ mice (n = 7) exhibited acinar cell atrophy (Figure 9*B*), inflammatory cells, dilated ducts, and fatty replacement. At 12 weeks of age, all *T7D23A*^het^ mice (n = 8) progressed to CP, and all *T7D23A*^het^ × *Spink1-KO*^het^ mice had severe CP with increased acinar cell loss relative to *T7D23A*^het^ animals (Figure 9*B*).

**Figure 10:**
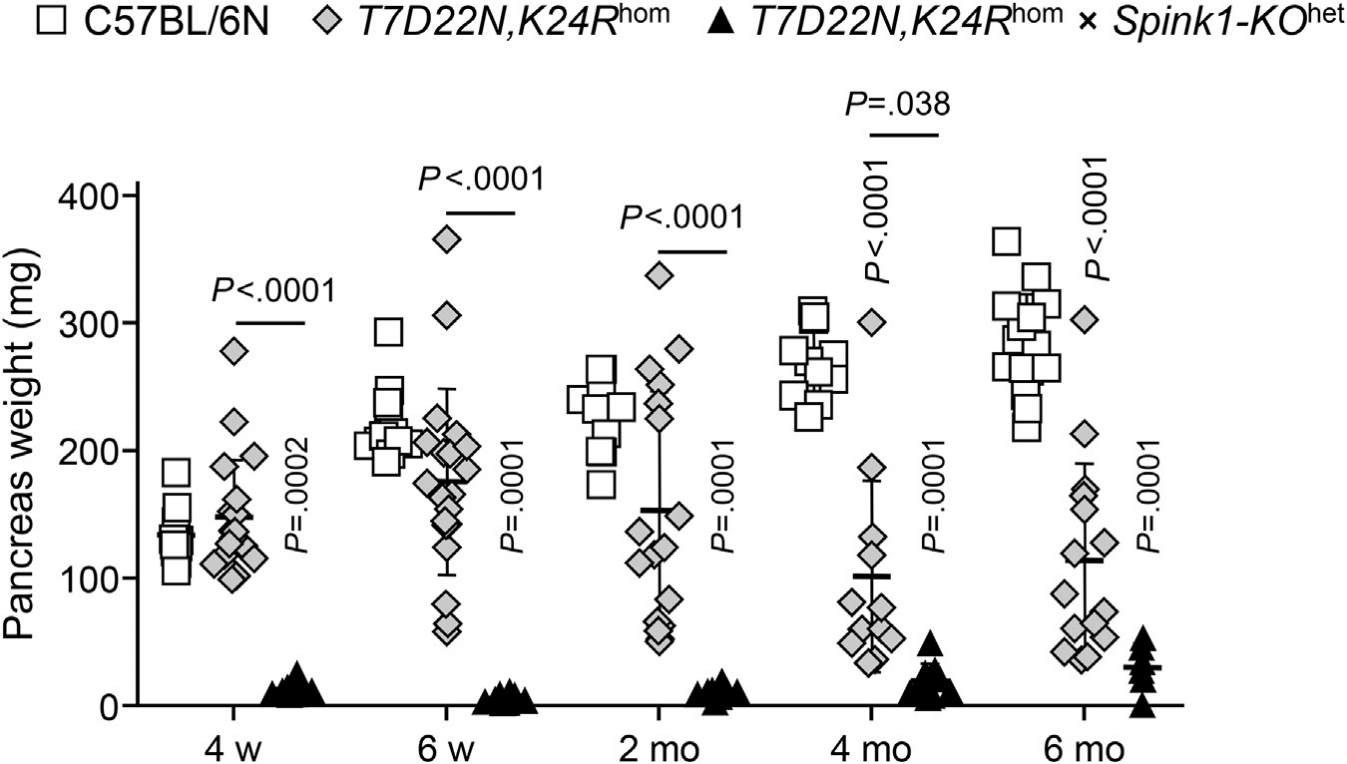
**Effect of heterozygous *Spink1* deletion on chronic pancreatitis severity in *T7D22N,K24R***^**hom**^**mice.** C57BL/6N, *T7D22N,K24R*^hom^, and *T7D22N,K24R*^hom^ × *Spink1-KO*^het^ mice were euthanized at 4 weeks, 6 weeks, 2 months, 4 months, and 6 months of age, and the pancreas weight was measured to assess bulk acinar atrophy. Note that part of this experiment showing data for C57BL/6N and *T7D22N,K24R*^hom^ mice was published previously.^28^ Individual values with the mean and standard deviation were graphed. The difference of means was analyzed by 1-way analysis of variance followed by Tukey’s post-hoc test.

**Figure 11:**
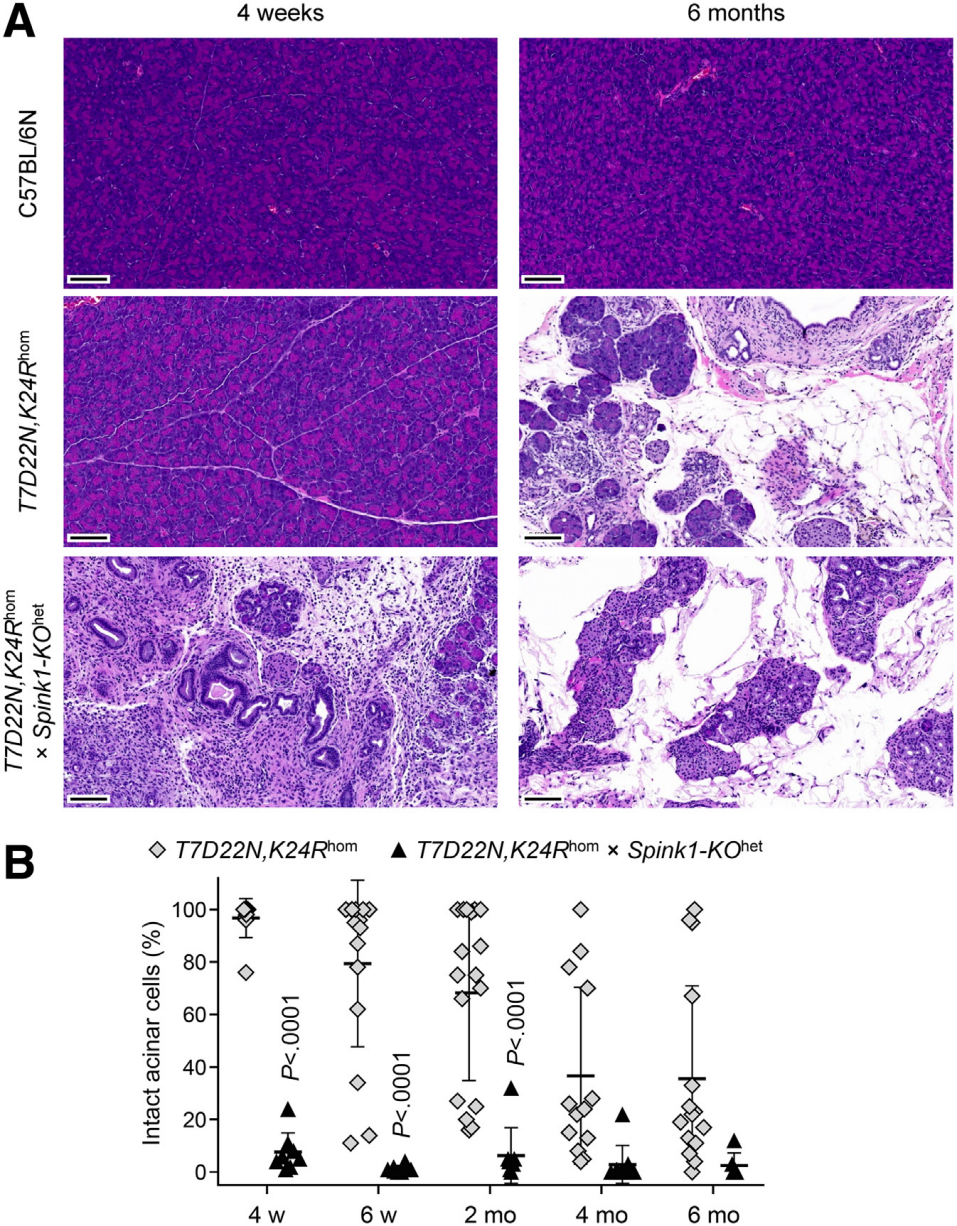
**Histological evaluation of chronic pancreatitis severity in *T7D22N,K24R***^**hom**^**× *Spink1-KO***^**het**^**mice.** C57BL/6N, *T7D22N,K24R*^hom^, and *T7D22N,K24R*^hom^ × *Spink1-KO*^het^ mice were euthanized at 4 weeks, 6 weeks, 2 months, 4 months, and 6 months of age. (*A*) Representative hematoxylin-eosin stained pancreas sections from 4-week-old (*left panels*) and 6-month-old (*right panels*) mice. Scale bars are 100 μm. (*B*) Histology scoring for intact acinar cells. Individual values with the mean and standard deviation were plotted. The difference of means was analyzed by 1-way analysis of variance followed by Tukey’s post-hoc test.

In a similarly dramatic fashion, *T7D22N,K24R*^hom^ × *Spink1-KO*^het^ mice developed severe CP as early as 4 weeks of age, as judged by the massive pancreas atrophy (Figure 10). The pancreas weight of C57BL/6N control mice increased as a function of time and almost doubled by 6 months of age relative to the 4-week value. *T7D22N,K24R*^hom^ mice exhibited decreasing pancreas weight after 2 months of age, as CP with acinar atrophy set in. In contrast, the weight of the pancreas from *T7D22N,K24R*^hom^ × *Spink1-KO*^het^ mice was severely reduced as early as 4 weeks of age and remained diminished throughout the 6-month time course. Histology analysis confirmed the early-onset CP in *T7D22N,K24R*^hom^ × *Spink1-KO*^het^ mice relative to *T7D22N,K24R*^hom^ animals. As shown in Figure 11*A*, at 4 weeks of age, the pancreas of *T7D22N,K24R*^hom^ mice looked normal, indistinguishable from those of C57BL/6N, whereas *T7D22N,K24R*^hom^ × *Spink1-KO*^het^ mice showed early CP characterized by massive acinar atrophy, high number of inflammatory cells, and acinar-to-ductal metaplasia. At 6 months of age, end-stage chronic disease was evident in both strains with large areas replaced by adipose tissue (Figure 11*A*). When multiple pancreas sections from mice euthanized at various ages were scored for intact acinar cells, the results clearly confirmed the drastic, early-onset acinar cell atrophy in *T7D22N,K24R*^hom^ × *Spink1-KO*^het^ mice relative to *T7D22N,K24R*^hom^ mice, which showed slowly progressing disease with more individual variation (Figure 11*B*). Taken together, the findings indicate that *Spink1* deficiency strongly promoted disease onset and progression in mouse strains with trypsinogen mutations that are prone to develop spontaneous CP in a trypsin-dependent manner.

## Discussion

In the present study, we set out to demonstrate that heterozygous *Spink1* deficiency increases severity of trypsin-dependent CP in mice. Heterozygous *SPINK1* mutations are strong risk factors for CP in humans; however, animal models to date failed to offer direct evidence for this notion. Here, we utilized not only the classic cerulein-induced pancreatitis model but also recently developed trypsinogen mutant mouse strains.

In agreement with previous reports,^13^^,^^16^ we found that the severity of cerulein-induced acute pancreatitis was unaffected by heterozygous *Spink1* deletion when the disease was elicited with transient hyperstimulation (10 hourly injections). Although this finding may have been baffling at the time of the earlier reports, recent developments in the field offer better understanding. Thus, in this model of secretagogue-induced acute pancreatitis, the pathological responses are not mediated by intrapancreatic trypsin activity.^32^ Consequently, severity parameters should be unaffected by the extent of SPINK1-mediated trypsin inhibition. Furthermore, we also made the somewhat unexpected observation that intrapancreatic trypsin and chymotrypsin activities induced by a single injection of cerulein were unchanged in *Spink1-KO*^het^ mice relative to C57BL/6N controls. It appears that in the vesicular compartment where cathepsin B generates trypsin activity, SPINK1 is ineffective, possibly due to degradation. Our results support the notion that transient cerulein hyperstimulation causes trypsin-independent acute pancreatitis where heterozygous loss of *Spink1* has no impact on severity.

Interestingly, a different picture emerged when mice were subjected to prolonged hyperstimulation with cerulein (2 × 8 hourly injections). Under these conditions, acute pancreatitis was significantly more severe in *Spink1-KO*^het^ mice relative to C57BL/6N animals, and intrapancreatic trypsin and chymotrypsin activities were also significantly higher in the *Spink1*-deficient mice when measured 30 minutes after the last injection. These observations indicate that under prolonged hyperstimulation with cerulein, acute pancreatitis becomes significantly more trypsin-dependent, and protective SPINK1 levels can have a measurable impact on disease severity.

Using mice carrying rapidly autoactivating trypsinogen mutants, we previously demonstrated that after an acute episode of cerulein-induced pancreatitis, progressive disease develops characterized by CP-like features such as acinar atrophy, macrophage infiltration, acinar-to-ductal metaplasia, duct dilation, fibrosis, and adipose tissue replacement.^28^^,^^29^ Recovery is slow and incomplete; even 3 months after the acute episode, the pancreas is not fully normal. In contrast, C57BL/6N mice recover rapidly and show essentially normal histology within 3 days. This progressive CP phenotype is driven by intrapancreatic trypsin activity and can be prevented by a small-molecule trypsin inhibitor administered orally after the cerulein injections.^25^^,^^30^ Remarkably, *Spink1-KO*^het^ mice exhibited a similar phenotype, which was observed after 10 injections of cerulein and even more strongly after 2 × 8 injections. Importantly, the CP-like disease was associated with elevated intrapancreatic trypsin activity in *Spink1-KO*^het^ mice relative to wild-type controls. These results indicate that heterozygous deletion of *Spink1* facilitates the development of trypsin-dependent CP after cerulein-induced acute pancreatitis.

The strongest evidence that heterozygous *Spink1* deficiency promotes CP came from the experiments using the *T7D23A* and *T7D22N,K24R* trypsinogen mutant strains. *T7D23A*^het^ and *T7D22N,K24R*^hom^ mice develop CP spontaneously, which progresses within months to an atrophic pancreas with fatty replacement of acinar cells.^22^^,^^28^ When *T7D23A*^het^ mice were crossed with *Spink1-KO*^het^ mice, the disease course accelerated, and atrophy was so pronounced that the pancreas dysfunction resulted in measurable body weight loss. The effect of the *Spink1-KO*^het^ allele was even more dramatic in *T7D22N,K24R*^hom^ mice, which normally exhibit CP with a later age of onset and slower progression. However, heterozygous *Spink1* deletion resulted in severe pancreas atrophy as early as 4 weeks of age. Although data are not shown, we also crossed the *Spink1-KO*^het^ strain with heterozygous *T7D22N,K24R*^het^ mice, which do not develop CP spontaneously.^28^ Interestingly, 4 of 11 compound heterozygous mice had CP at 6 months of age, indicating that intrapancreatic trypsinogen activation was increased to pathological levels in *T7D22N,K24R*^het^ mice by the heterozygous deletion of *Spink1*.

It is noteworthy that reduced SPINK1 levels modify disease severity without causing spontaneous pancreatitis. In fact, early genetic literature referred to human *SPINK1* variants as disease modifiers in CP.^6^ The situation is somewhat reminiscent of how lipolysis worsens acute pancreatitis but does not initiate the disease, as documented in a number of recent studies.^33^, ^34^, ^35^

While this paper was under review, a new study was published where adeno-associated virus was used as a vector to overexpress human SPINK1 in the mouse pancreas.^36^ The authors demonstrated protective effects in acute and chronic pancreatitis induced by cerulein injections or duct ligation, and in the c.194+2T>C *Spink1* model. A direct comparison with the present study is difficult due to the different experimental conditions employed, but the overall efficacy of virally transduced SPINK1 against pancreatitis is consistent with our conclusions.

Taken together, the findings presented here provide compelling evidence that heterozygous *Spink1* deficiency increases severity and rate of progression of CP in trypsin-dependent mouse models. Thus, heterozygous *Spink1* deletion in mice reproduces the loss-of-function effects of human heterozygous *SPINK1* mutations and confirms that even partial reduction in protective SPINK1 levels can increase intrapancreatic trypsin activity and promote CP.

## Methods

### Accession Numbers and Nomenclature

*Spink1* (legacy name *Spink3*) genomic sequence: NC_000084.7, *Mus musculus* strain C57BL/6J chromosome 18, GRCm39. *Spink1* coding DNA: NM_009258.5, *Mus musculus* serine peptidase inhibitor, Kazal type 1 (*Spink1*), mRNA.

### Animal Studies Approval

Animal experiments were performed at the University of California, Los Angeles (UCLA) with the approval and oversight of the Animal Research Committee, including protocol review and post-approval monitoring. The animal care program is managed in full compliance with the United States (U.S.) Animal Welfare Act, the U.S. Department of Agriculture Animal Welfare Regulations, the U.S. Public Health Service Policy on Humane Care and Use of Laboratory Animals, and the National Research Council's Guide for the Care and Use of Laboratory Animals. UCLA has an approved Animal Welfare Assurance statement on file with the U.S. Public Health Service, National Institutes of Health, Office of Laboratory Animal Welfare, and it is accredited by the Association for Assessment and Accreditation of Laboratory Animal Care International (AAALAC).

### Generation of the Spink1-KO Mouse Strain

The gene encoding mouse *Spink1* (legacy name *Spink3*) is located on chromosome 18, it spans ∼9 kb, and comprises 4 exons. *En bloc* deletion of the *Spink1* gene (*Spink1-KO*) from c.-473 to c.∗1737 (11,200 nucleotides) was accomplished by CRISPR/Cas9-mediated genome engineering (Cyagen US Inc). *Spink1-KO* mice were maintained on the C57BL/6N genetic background in the heterozygous state (*Spink1-KO*^het^) because homozygous animals died shortly after birth. C57BL/6N mice obtained from Charles River Laboratories or produced in our breeding facility from the same stock were used as experimental controls. Both male and female animals (8–10 weeks of age) were included in the studies.

### Genotyping Spink1-KO^het^ Mice

The following primers were used to genotype mice for the *Spink1-KO* allele. Common forward primer F1, 5′-CAA ACA CCC TGG GAA AGA TAA TCT GTC-3′, wild-type allele reverse primer R1, 5′-TAC CTG CTA AAC TCA GCA GGG CC-3′, deletion allele reverse primer R2, 5′-GGC CTG TCC AGG GAT CAA TCA TAA-3′. PCR reactions contained all 3 primers. The amplicon size from the deletion allele was 540 bp, whereas the wild-type allele gave an 839 bp product.

### Trypsinogen Mutant Mouse Strains

Development and characterization of *T7D23A* and *T7D22N,K24R* mice carrying rapidly autoactivating cationic trypsinogen (isoform T7) mutants in the C57BL/6N genetic background were reported recently.^22^^,^^28^ *T7D23A* mice were maintained in the heterozygous state (*T7D23A*^het^) because homozygous animals become runted and die early. *T7D22N,K24R* mice were kept in the homozygous state (*T7D22N,K24R*^hom^).

### Western Blot and Densitometry

Mouse pancreas tissue (30–40 mg) was homogenized in 300- to 400-μL ice-cold PBS (pH 7.4) supplemented with Halt protease and phosphatase inhibitor (catalog number 1861284, Thermo Fisher Scientific). The homogenate was cleared by centrifugation (10 minutes, 13,500 rpm, 4 °C) and aliquots of the supernatant (30 μg total protein per lane) were electrophoresed on 15% SDS-PAGE minigels. The proteins were transferred onto an Immobilon-P membrane (catalog number IPVH00010, MilliporeSigma) and the membrane was blocked with 10% solubilized milk powder. Incubations with primary and secondary antibodies were carried out overnight at 4 °C and for 1 hour at 22 °C, respectively. Membranes were first probed for SPINK1 or T7 trypsinogen, then stripped, and re-probed for ERK1/2. The following antibodies were used. Rabbit polyclonal antibody against mouse SPINK1 was used at a dilution of 1:1,000 (catalog number 2744, Cell Signaling Technology); rabbit polyclonal antibody against T7 trypsinogen^22^ was used at 1:10,000 dilution; rabbit monoclonal antibody against ERK1/2 was used at 1:1,000 dilution (catalog number 4695, Cell Signaling Technology). As secondary antibody, horseradish peroxidase-conjugated goat anti-rabbit IgG (catalog number HAF008, R&D Systems) was used at 1:10,000 dilution. T7 trypsinogen and ERK1/2 were detected with the SuperSignal West Pico chemiluminescent substrate (catalog number 34580, Thermo Fisher Scientific). SPINK1 protein was detected with the SuperSignal West Femto Maximum Sensitivity chemiluminescent substrate (catalog number 34095, Thermo Fisher Scientific). Densitometric evaluation of protein band intensities on Western blots was carried out with the ImageJ software (version 1.52a).

### RNA Isolation and Reverse Transcription qPCR

Total RNA was isolated from mouse pancreata (20 mg) using the RNeasy Plus Mini Kit (Qiagen). Two μg RNA was reverse-transcribed with the High Capacity cDNA Reverse Transcription Kit (catalog number 4368814, Thermo Fisher Scientific). Quantitative PCR was performed with the Mm00436765_m1 (*Spink1*, FAM dye) and Mm01612987_g1 (*Rpl13a* reference gene, VIC dye) TaqMan probes, using the Taqman Universal PCR Master Mix (catalog number: 4304437, Thermo Fisher Scientific). Relative expression levels were estimated with the ΔΔCT method as described before.^23^

### Cerulein-induced Pancreatitis

To induce acute pancreatitis, mice were administered hourly injections of cerulein (50 μg/kg dose) intraperitoneally causing transient (10 injections) or prolonged (2 × 8 injections on consecutive days) hyperstimulation, as indicated. Control mice were given saline injections. To assess acute pancreatitis, mice were euthanized 1 hour (10 injections) or 30 minutes (2 × 8 injections) after the last injection. To study progression to CP after the acute episode, mice were euthanized 1 week (10 injections) or 3 days (2 × 8 injections) after the last injection.^31^

### Intrapancreatic Protease Activity

Intrapancreatic trypsin and chymotrypsin activity were measured from freshly prepared pancreas extracts according to our published protocol.^37^ Mice were euthanized 30 minutes after a single injection of cerulein (50 μg/kg dose), 30 minutes after 2 × 8 cerulein injections, or 3 days after 2 × 8 cerulein injections, as indicated. Control mice were given saline injections and were euthanized at the same times. Enzyme activity was expressed in RFU/sec/mg protein units, which represents the rate of substrate cleavage in relative fluorescent units per second, normalized to the total protein content.

### Plasma Amylase Assay

Heparinized blood plasma (1 μL) was used to measure amylase activity with a kinetic assay, as described previously.^23^ The rate of substrate cleavage was expressed in mOD/min unit.

### Histology

Pancreas tissue was fixed and stored in 10% neutral buffered formalin, paraffin-embedded, sectioned, and stained with hematoxylin-eosin (UCLA Translational Pathology Core Laboratory and Department of Pathology and Laboratory Medicine, Cedars-Sinai Medical Center). Pancreas sections of cerulein-treated mice were scored by visual inspection for 3 parameters of acute pancreatitis (tissue edema, inflammatory cell infiltration, and acinar cell necrosis) and for intact acinar cells in CP. Pancreas sections from saline-treated mice were not scored because they exhibited no histological changes. Scoring was performed in a blinded fashion by 2 independent observers, and their results were averaged. Pancreas sections were scanned with the Pannoramic DESK II DW slide scanner (3DHISTECH) and the files were viewed with the SlideViewer (version 2.6, 3DHISTECH) software. Using the 1.5× magnification setting, the entire tissue was visualized and divided into 10 equal squares with the “Annotation” feature. The squares covered about 75% to 80% of the total tissue area. All 10 squares were scored, and the average was used as the final score for a given slide. Edema, inflammatory cells, and intact acini in CP were scored using the 10× magnification setting while acinar cell necrosis was scored at 10 to 20×.

To estimate the degree of tissue edema, an arbitrary scale of 0 to 3 was used, where 0 indicated the absence of visible edema. A score of 1 denoted slight widening of the inter-lobular spaces, corresponding to <10% of the area inspected, with no significant separation of acini due to inter-acinar edema. A score of 2 specified moderate widening of the inter-lobular spaces, corresponding to 10% to 50% of the area analyzed, with slight but visible separation of acini due to inter-acinar edema. A score of 3 indicated marked widening of the inter-lobular spaces, corresponding to >50% of the visual area, together with significant separation of acini due to inter-acinar edema. In cases where the degree of tissue edema was not uniform within a given square, scores of 0.5, 1.5, and 2.5 were used, as appropriate.

To evaluate the extent of inflammatory cell infiltration, an arbitrary scale of 0 to 3 was used, where 0 indicated the absence of visible inflammatory cells. A score of 1 denoted the presence of low-density infiltrates (<5 cells per 0.01 mm^2^ area) in the inter-lobular spaces, corresponding to <10% of the area analyzed, with no inflammatory cells around the acini. A score of 2 indicated medium-density infiltrates (5–20 cells per 0.01 mm^2^ area) within the inter-lobular spaces observed in 10% to 50% of the visual field, with some inflammatory cells visible in the inter-acinar spaces. A score of 3 specified high-density infiltrates (>20 cells per 0.01 mm^2^ area) in the widened inter-lobular spaces visible in >50% of the tissue area inspected. In cases where the inflammatory cell infiltration was not uniform within a given square, scores of 0.5, 1.5, and 2.5 were used, as appropriate.

The degree of acinar cell necrosis in acute pancreatitis and the proportion of intact acinar cells in CP were expressed as percent of the total tissue area examined. Necrosis was defined as partial or complete loss of acinar cell histoarchitecture, including areas with amorphous, pink material, acinar cell ghosts, and swollen, vacuolated acinar cells.^32^

### Statistics

Results were graphed as individual data points with the mean and standard deviation values indicated. The difference of means between 2 groups and within multiple groups was analyzed by 2-tailed unpaired *t*-test and by 1-way analysis of variance with Tukey’s post-hoc analysis, respectively. *P* < .05 was considered statistically significant.
